# Validation of Novel Metrics from the Accommodative Dynamic Profile

**DOI:** 10.3390/vision2030034

**Published:** 2018-08-21

**Authors:** Nicola Szostek, Hetal Buckhurst, Christine Purslow, Thomas Drew, Avril Collinson, Phillip Buckhurst

**Affiliations:** 1 Eye and Vision Research Group, School of Health Professions, University of Plymouth, Plymouth PL4 8AA, UK; 2 School of Optometry and Vision Sciences, Cardiff University, Cardiff CF24 4HQ, UK; 3 Ophthalmic Research Group, School of Life and Health Sciences, Aston University, Birmingham B4 7ET, UK

**Keywords:** accommodative dynamics, accommodative facility, auto-refraction, accommodative latency, response times, time for accommodative change

## Abstract

Objective and subjective methods of assessing time taken for accommodative change (ToAC) include accommodative dynamics (AD) and accommodative facility (AF). This study investigates the validity of novel metrics derived from the AD-profile and explores their relationship with AF. AD were assessed using a modified open-field autorefractor in 43 healthy adults. Non-linear regression curves were fitted to the data to derive: latency-of-accommodation (nLoA) and -disaccomodation (nLoD), Time-for-accommodation (ToA) and -disaccommodation (ToD), and objective-ToAC (oToAC). Latencies were also calculated through visual inspection of the AD data as in previous studies (pLoA and pLoD). AF was used to assess subjective-ToAC. Statistical analysis explored the relationships between the AD-metrics and AF. Subjects were assessed on three visits to examine intra- and inter-observer repeatability. nLoA and nLoD were greater than pLoA (*p* = 0.001) and pLoD (*p* = 0.004) respectively. nLoA and nLoD also demonstrated greater intra- and inter-observer repeatability than pLoA and pLoD. AF demonstrated a moderate, inverse correlation with ToA (*p* = 0.02), ToD (*p* = 0.007), and oToAC (*p* = 0.007). ToD was the single best accommodative predictor of AF (*p* = 0.011). The novel method for deriving latency was more repeatable, but not interchangeable with the techniques used in previous studies. ToD was the most repeatable metric with the greatest association with AF.

## 1. Introduction

Accommodation is the dioptric power change of the eye in response to a near target [1], which is brought about by an increase in the surface curvature and a decrease in equatorial diameter of the crystalline lens [2,3,4]. Accommodative function can be quantified by numerous parameters including the speed, accuracy, amplitude and sustainability of the response. These accommodative parameters can be assessed by a variety of subjective and objective methods, as summarised in Table 1.

Subjective methods that assess accommodative parameters rely on the patient’s perception of blur as an end point criterion; hence, these measurements are a combined value of both true- and pseudo- accommodation. Objective methods of assessing accommodation measure the refractive status of the eye whilst viewing a target at a set distance; hence, the end-point criterion is entirely objective, removing the component of pseudo-accommodation [1]. Indeed, previous studies that have compared objective and subjective measures of the accommodative amplitude observed that subjective techniques produced higher and more variable values for accommodation than their objective counterparts [1,5,6,7,8,9,10,11,12,13,14]. The accommodative facility test (AF) is a subjective technique which is used to assess the speed and accuracy of the eyes’ ability to change focus from distance-to-near and near-to-distance [15]. Clinically, the AF test is used to investigate symptomatic accommodative dysfunction [16,17,18], and is predominantly used in a paediatric setting [19,20]. During the clinical evaluation of AF, the number of times (cycles) a change in accommodation can be achieved through an alternating dioptric stimulus (of 4.00 DS) within 1 min is assessed [15,21].

**Figure 1:**
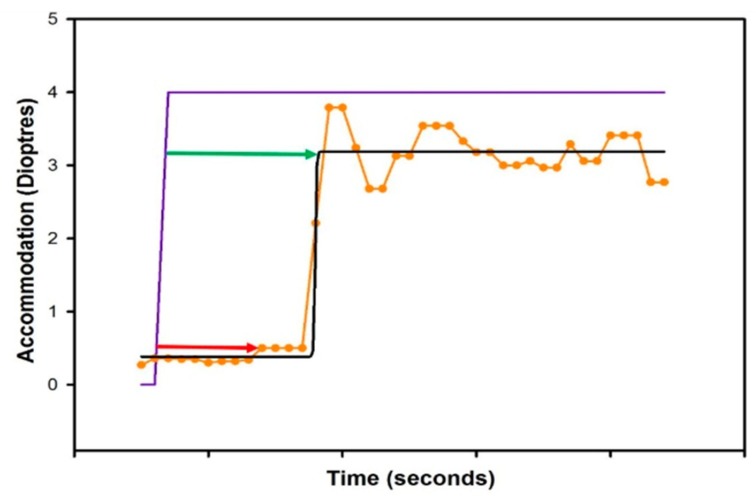
An accommodative dynamic profile of a pre-presbyope. The purple line represents the onset of the accommodative stimulus, the yellow line represents the accommodative response and the black line is the curve fitted to smooth the data. The red arrow demonstrates the latency of accommodation [28] and the green arrow represents the response times or time for accommodation/response time [24].

Dynamic assessment of accommodation is an objective measure of accommodative parameters utilizing either auto-refractors, power-refractors or abberometers. Numerous objective metrics can be derived from accommodative profiles to describe the parameters, as shown in Table 1. These metrics can include: absolute or the magnitude of the accommodative response, accommodative lag, time constants, response times, peak velocity of accommodation, and microfluctuations [4,8,9,14,22,23,24]. Figure 1 illustrates, and Table 2 describes the metrics used to quantify the time taken to accommodate in more detail, and the various methods that have been used to derive these from the accommodative dynamic profile.

Understanding the relationships between the metrics derived from the accommodative profile and their corresponding subjective and objective clinical tests is important for assessing the clinical significance of findings from research studies. Numerous studies have examined the relationships between the accuracy or absolute accommodative response, to the corresponding clinical tests [7,8,9,10,25]; however, comparatively fewer have examined the relationship between the metrics used to describe the time metrics which include accommodative latency, time constants, peak velocity of accommodation, and response times, with the corresponding subjective test of AF [4,24].

As described in Table 2, significant variability exists in the methods used to define, measure, and calculate each time metric from the accommodative dynamic profile [4,8,14,22,23,24,26]. Additionally, prior to deriving each metric, it is common to smooth the data from the accommodative dynamic to remove erroneous results [4,8,14,22,23,24]. Different methods to achieve this have been applied, including averaging three consecutive points and then re-plotting the profile [14], or through non-linear regression analysis [4,8,23,24,27]. This lack of standardisation and clarity in deriving time metrics in previous literature presents difficulties in replicating studies and comparing results. Therefore, the aim of this study was to present novel methods for deriving the latency of accommodation and the time for accommodative change, with clearly defined objective end points, removing the need for subjective visual inspection of the data. Furthermore, the relationship between these novel metrics with AF and age is also explored.

## 2. Materials and Methods

Forty-three subjects (18 males, 25 females) with a mean spherical equivalent refractive error of RE: −0.90 DS (SD = 2.10) (range: −10.00 DS to +1.38 DS) and LE: −0.88 DS (SD = 2.00) (range: −9.50 DS to +3.75 DS) of a mean age 31 ± 8 years (range 19 to 48 years) were recruited. The exclusion criteria included current or previous ocular pathologies or trauma, binocular vision abnormalities and diabetes mellitus [29]. The study adhered to the tenets of the Declaration of Helsinki and was approved by the University of Plymouth’s Research Ethics Committee. All subjects gave informed consent to participate in the study.

Auto-refraction was conducted using a Grand Seiko Auto-refractor WAM-5500 (Grand Seiko Co. Ltd., Hiroshima, Japan), followed by a subjective refraction to establish any habitual refractive error. Refractive error > ± 0.50 DS and/or >0.75 DC was corrected with daily soft contact lenses. Subjects whose corrected visual acuity (VA) was worse than 0.0 LogMAR (6/6) were excluded at this stage.

Each subject was assessed on three separate visits to evaluate intra- and inter-observer repeatability. At visit one and two, a single examiner assessed each subject, whilst at the third visit, a second examiner who was blind to the previous results examined each subject. To minimize order effects the accommodation measurements were conducted in a random order.

### 2.1. Accommodative Facility (AF)

AF was assessed monocularly with the contralateral eye occluded. The subject was presented with a four letter N5 target at a viewing distance of 40 cm. Using confirmation flippers, a +2.00 DS lens was presented in front of the viewing eye and the subject was asked to report when the target first became clear. Once a positive response was given, the flippers were rotated so that a −2.00 DS lens was presented, the subject was again asked to report verbally when the target was clear. Presentation of the +2.00 DS lens followed by the −2.00 DS lens was classed as one cycle, representing a total accommodative change of 4.00 DS. After an initial ‘practice’ with two cycles or until the test was understood, the number of full cycles achieved within 1 min was recorded.

### 2.2. The Accommodative Dynamic Profile

**Figure 2:**
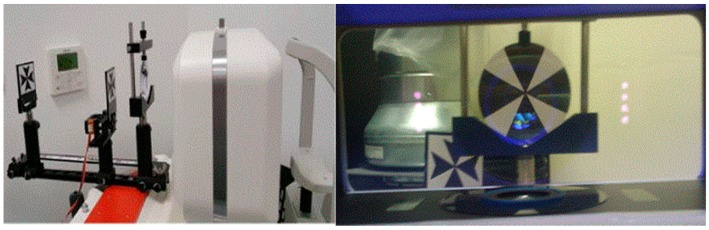
The set-up of the auto-refractor and Badal lens system (**left**), and the subject’s view of the Maltese targets whilst measuring accommodative dynamics (**right**).

The accommodative dynamic profile was assessed monocularly using the Grand Seiko Auto-refractor WAM-5500 with a motorised DynaWAM Badal adaption (Figure 2, left). The DyanWAM Badal adaption consisted of a 5.00 DS lens and two maltese crosses, one fixed at a distance of 20 cm (with an accommodative demand of 0 Dioptres (D)) from the Badal lens, and the second, which is motorized to ‘flip’ into and out of the viewing plane just behind the Badal lens (with an accommodative demand of 4 D). With the contralateral eye occluded, subjects viewed a Maltese cross target within the Badal lens system (Figure 2, right). Subjects were instructed to focus on the centre of whichever Maltese cross was closest to them, and to ‘make it as clear as possible’. Once the DynaWAM Badal system and auto-refractor were activated, real time measurements of refractive status whilst the accommodative demand was alternated between 0 D and 4 D were captured, at a rate of 8 Hz for six full cycles of accommodation/disaccommodation. During the first cycle the accommodative response was monitored by the observer to ensure the subject was able to perform the task; this cycle was subsequently discounted from analysis for all subjects. Any subjects who did not demonstrate an accommodative response during the first cycle were excluded from data analysis.

### 2.3. Data Analysis

**Figure 3:**
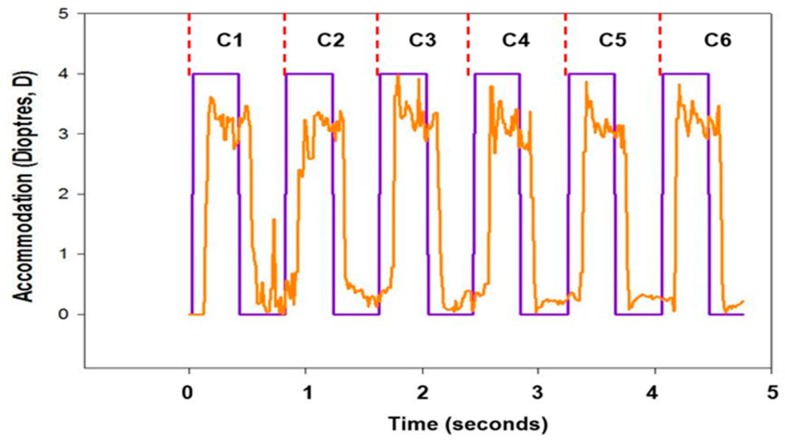
The accommodative response of a 27-year-old participant (yellow line), over six cycles of accommodative demand flipping between 0 D and 4 D (purple line).

Data were exported from the DynaWAM software to an Excel spreadsheet (Excel 2013, Microsoft Office, Redmond, Washington, USA), where each accommodative dynamic profile (Figure 3) was visually inspected and split into individual cycles (labelled C1 to C6). Both C1 and C6 were deemed unreliable due to time delays in starting and terminating the auto-refractor and DynaWAM software; therefore, only C2 to C5 were used for analysis. Cycles were further split into accommodation and disaccommodation; accommodation was defined as the phase between the first to the last data point with the 4 D stimulus. The disaccommodation phase was defined as the first to the last data point with the 0 D stimulus.

Matlab software (R2014a, The MathWorks Inc., Natick, MA, USA) was utilised to fit a 4-parameter non-linear sigmoidal regression curve (Equation (1)) to the accommodative response data points in each cycle.

(1) $$y=a+\left\{\frac{b}{1+e\;\left(-\left[\frac{x-d}{c}\right]\right)}\right\}$$

Equation (1): A 4-parameter non-linear regression where ***a*** is the minimum accommodative response (diopters), ***b*** = the asymptote, ***c*** is the mid-point between the minimum and maximum accommodative response (diopters), ***y*** is accommodative response (dioptres) and ***x*** is the time (seconds).

Erroneous measurements that resulted from a subject blinking were identified and removed; if a blink occurred during accommodation or disaccommodation, the data from that curve were excluded from analysis. The three cycles with the most significant linear fits of r_2_ were used to derive the following metrics.

### 2.4. Accommodative Latency

Latency of accommodation and disaccommodation were calculated using a method similar to that used in previous studies (*p*) (pLoA and pLoD) [28]. Data smoothing was conducted as described by Anderson et al. (2010) [14]. The data was then visually inspected to identify the first data point corresponding to the initial accommodative response [28].

For the purposes of this study a novel method for deriving latency of accommodation and disaccommodation (nLoA and nLoD respectively) was calculated using Matlab software:

- nLoA was defined as the time taken to achieve 1% of the full accommodative response to the 4 D near target.
- nLoD was defined as the time taken to achieve 1% of the full disaccommodative response once the 4 D target was removed and 0 D stimulus introduced.

The criterion of 1% of the full accommodative/disaccommodative response was chosen as this presented an objective starting point of the accommodative response.

Equation (2) was used to calculate nLoA and nLoD.

(2) $$y=a+\left\{\frac{\left(b\times 0.01-a\right)}{1+e\;\left(-\left[\frac{x-d}{c}\right]\right)}\right\}$$

Equation (2): Calculation of nLoA and nLoD where ***a*** is the minimum accommodative response (diopters), ***b*** = the asymptote, ***c*** is the mid-point between the minimum and maximum accommodative response (diopters), ***y*** is the accommodative response (dioptres) and ***x*** is the time (seconds).

### 2.5. Time for Accommodation (ToA) and Disaccommodation (ToD), and Time for Accommodative Change (ToAC)

ToA was defined as the time taken to achieve 99% of the full accommodative response to the 4 D near target. ToD was defined as the time taken to achieve 99% of the full disaccommodative response once the 4 D target was removed and 0 D stimulus introduced. The objective ToAC (oToAC) was defined as the sum of ToA and ToD. 99% of the full accommodative/disaccommodative response was chosen as the end point to correspond with 1% as the starting point.

Equation (3) was used to calculate ToA and ToD.

(3) $$y=a+\left\{\frac{\left(b\times 0.99-a\right)}{1+e\left(-\left[\frac{x-d}{c}\right]\right)}\right\}$$

Equation (3): Calculation of ToA and ToD where ***a*** is the minimum accommodative response (diopters), ***b*** = the asymptote, ***c*** is the mid-point between the minimum and maximum accommodative response (diopters), ***y*** is the accommodative response (dioptres) and ***x*** is the time (seconds).

The subjective time for accommodative change (sToAC) was the time taken (in seconds) to complete a single AF, this was calculated by dividing 60 s by the total number of cycles achieved in one minute.

### 2.6. Statistical Analysis

After visual inspection of descriptive statistics, histograms, box-plots and Shapiro-Wilks tests, all of the accommodation metrics were found to have a non-normal distribution. Wilcoxon’s Signed Rank tests were used to compare the accommodative metrics obtained for the right and left eye. As no significant difference was found at the 0.05 level, data from the right eye was used for further analysis. Intra- and inter-observer repeatability was examined by assessing Intraclass Correlation Coefficients (ICC) for visits 1–2 and visits 1–3, respectively. This was calculated using two-way mixed single measures (absolute agreement);

$$ICC\;\left(absolute,\;2\right)=\frac{subject\;variability}{\left(subject\;variability+measurement\;error\right)÷2}$$

Bland and Altman plots were constructed to examine the agreement and any proportional bias between nLoA-pLoA, nLoD-pLoD and sToAC-oToAC. To assess the associations and relationships between nLoA-pLoA, nLoD-pLoD and sToAC-oToAC, Spearman’s correlation coefficient and Wilcoxon’s signed-rank tests were also conducted.

Wilcoxon’s signed-rank tests were also conducted comparing nLoA-nLoD, pLoA-pLoD, and ToA-ToD to investigate whether the accommodation or disaccommodation metric was faster.

Multiple spearman’s Rho two-tailed tests were conducted to investigate the relationship between: (i) all accommodative metrics and mean spherical equivalent refractive error, (ii) the objective accommodative measurements and AF, and (iii) all accommodative metrics and age. Finally, both a forward stepwise and backward regression analysis were conducted to identify which metric was the best predictor of AF.

## 3. Results

**Figure 4:**
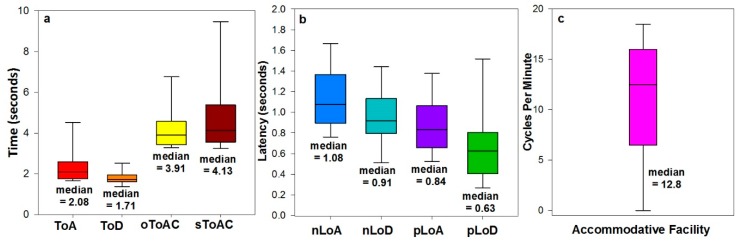
Box plots displaying the median, 10th, 25th, 75th and 90th percentiles of data for all accommodative metrics derived from the accommodative dynamic profile and accommodative facility.

The box plots of all the accommodative parameters measured are shown in Figure 4.

### 3.1. Comparison of Latency Calculation Metrics

**Figure 5:**
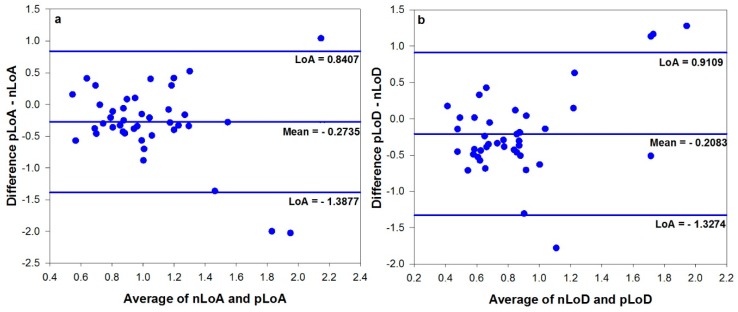
Bland and Altman plots of (**a**) novel Latency of Accommodation and previously used Latency of accommodation and (**b**) novel Latency of Disaccommodation and previously used Latency of Disaccommodation.

Latency values obtained using the metric nLoA and nLoD were greater than pLoA (*Z* = −3.212, *p* = 0.001) and pLoD (*z* = −2.920, *p* = 0.004) respectively. nLoA–pLoA and nLoD–pLoD also demonstrated moderate correlations (r_s_ = 0.342, *p* = 0.032 and r_s_ = 0.427, *p* = 0.004, respectively). The Bland and Altman plots comparing nLoA-pLoA and nLoD-pLoD (Figure 5) demonstrated a mean difference of 0.28 s and 0.24 s, respectively, with relatively large limits of agreement.

### 3.2. Correlations between Accommodative Metrics and Refractive Error

Mean sphere equivalent refractive error failed to demonstrate any significant association with the accommodative metrics (ToA: r_s_ = 0.114, *p* = 0.465; ToD: r_s_ = −0.100, *p* = 0.523; oToAC r_s_ = 0.046, *p* = 0.768; sToAC; r_s_ = 0.163, *p* = 0.341; nLoA r_s_ = 0.157, *p* = 0.316; nLoD r_s_ = 0.137, *p* = 0.380; pLoA r_s_ = 0.323, *p* = 0.85; pLoD r_s_ = −0.089, *p* = 0.577; AF r_s_ = −0.61, *p* = 0.697).

### 3.3. Correlations between Accommodative Metrics and AF

**Figure 6:**
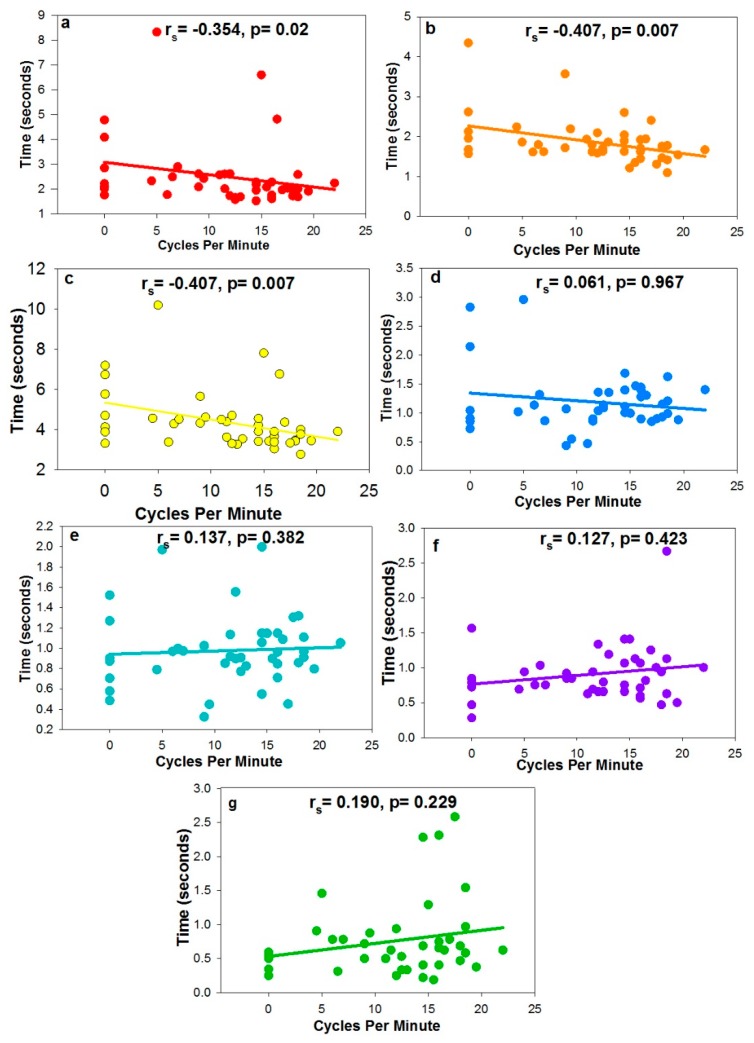
The correlation coefficient between AF and (**a**) Time for Accommodation, (**b**) Time for Disaccommodation, (**c**) Objective Time for Accommodative Change, (**d**) Novel Latency of Accommodation, (**e**) Novel Lactency of Disaccommodation, (**f**) Previously used Latency of Accommodation, and (**g**) Previously used Latency of Disaccommodation.

Figure 6 shows the correlations coefficients of the accommodative metrics when compared with AF. AF demonstrated a moderate, inverse correlation with ToA, ToD, and oToAC, but failed to demonstrate any significant association with nLoA, nLoD, pLoA, and pLoD.

### 3.4. Correlations between Accommodative Metrics and Age

**Figure 7:**
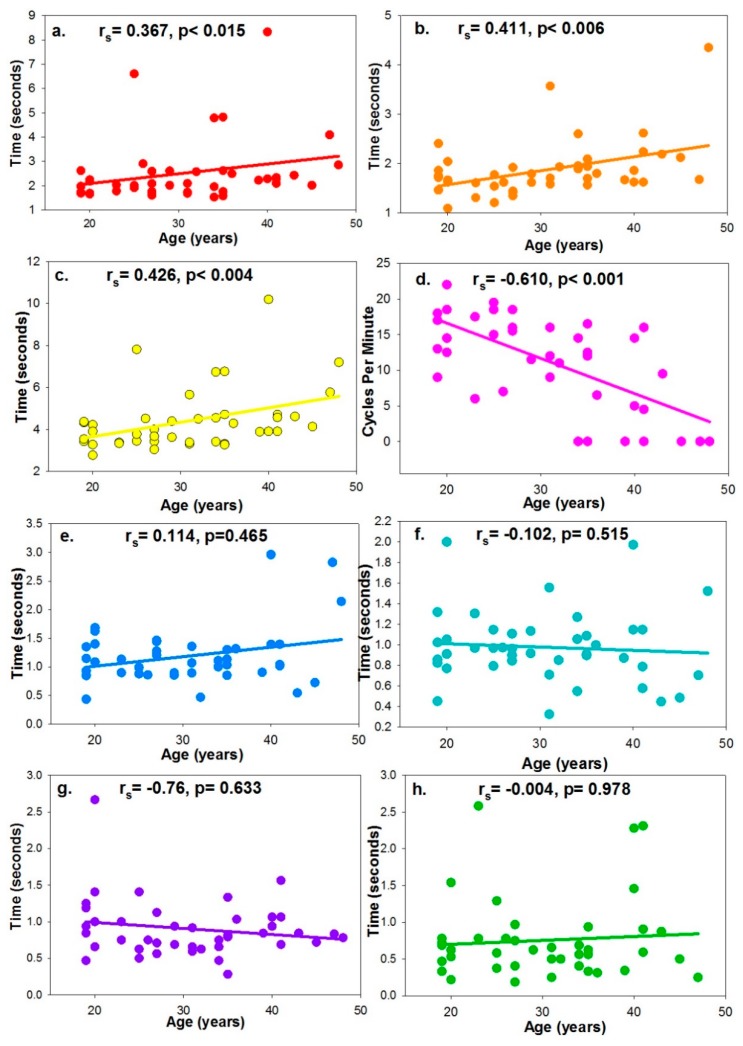
The correlation coefficient between age and (**a**) Time for Accommodation, (**b**) Time for Disaccommodation, (**c**) Objective Time for Accommodative Change, (**d**) Accommodative Facility, (**e**) Novel Latency of Accommodation, (**f**) Novel Latency of Disaccommodation (**g**) Latency of accommodation, and (**h**) Latency of Disaccommodation.

ToA, ToD and oToAC demonstrated a moderate, positive correlation with age, whereas AF showed a strong inverse correlation with age (Figure 7). However, none of the latency of accommodation or disaccommodation metrics were associated with age.

### 3.5. Comparison of oToAC and sToAC

**Figure 8:**
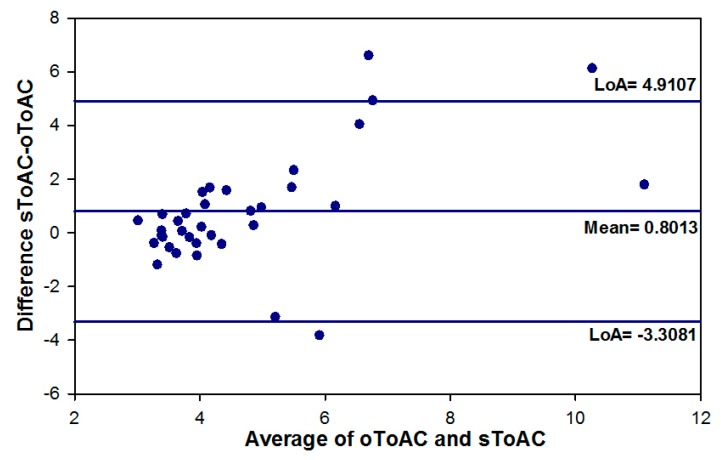
Bland and Altman plot of Subjective Time for Accommodative Change and Objective Time for Accommodative Change.

sToAC was significantly slower than oToAC (*z* = −2.498, *p* = 0.012) and the two metrics showed a moderate correlation (r_s_ = 0.371, *p* = 0.026). The Bland and Altman plot (Figure 8) revealed a mean difference of 0.80 s between the two metrics and significant proportional bias: As mean oToAC and sToAC times increased, the values for sToAC increased disproportionally to those for oToAC.

### 3.6. Repeatability

As shown in Table 3, AF demonstrated high levels of both intra-observer and inter-observer repeatability. The lowest level of intra-observer repeatability was found with ToA, and the lowest level of inter-observer repeatability was observed with pLoA.

### 3.7. Comparisons of Accommodation and Disaccommodation Metrics

All of the disaccommodative metrics were faster than the respective accommodative metrics: ToD < ToA: *Z* = −3.357, *p* < 0.001; nLoD < nLoA: Z= −3.236, *p* < 0.001; pLoD < pLoA: *Z* = −2.683, *p* = 0.007.

### 3.8. Regression Analysis

Both a forward stepwise and backward regression analyses were used as an exploratory test to determine which metric was the best predictor of AF. ToA, ToD and ToAC were the independent variables included, and it was found that ToD (β = −2.673, *p* = 0.011) was the single best predictor of AF. However, when age (β = −5.422, *p* < 0.001) was added into the model it was found to be the single best predictor of AF.

## 4. Discussion

The medians of nLoA and nLoD (1.07 s and 0.91 s, respectively) were higher than the medians of the previously used metrics pLoA and pLoD (0.84 s and 0.63 s respectively). These higher values could be attributed to the difference in methods of calculating latencies from the data. Firstly, the novel latency metrics presented in this study, nLoA and nLoD were derived by fitting a 4-paramenter non-linear regression curve; whereas pLoA and pLoD were attained by averaging three consecutive values. Secondly, nLoA and nLoD were calculated from the onset of the stimulus until 1% of the accommodative response was reached and it is conceivable that this would constitute a longer time period than pLoA and pLoD. Therefore, these are two similar, but separate metrics that are not interchangeable. Importantly, the novel metrics utilised objective curve fitting and an objective end point, rather than the visual inspection of data, and thus demonstrated greater repeatability.

Despite known differences in accommodative function between ametropic groups [30,31,32], the underlying mechanisms are poorly understood [32]. Interestingly, this study found no association with ametropia and the specific accommodative metrics examined; these observations corroborate the findings of previous studies [32,33,34].

The relationship between age and latency is equivocal; Anderson et al. (2010) found a significant decrease in both accommodative and disaccomodative latencies with age [14], whilst Kasthurirangan and Glasser (2006) observed an increase in disaccommodation with progressing age [8]. Conversely, others are in agreement with the present study and have found no correlation between age and latency [26,35,36,37]. It is conceivable that there is no detectable change in latency with age because accommodative latency is attributed to the time taken for the neurological processing involved in the recognition of a blurred target and the subsequent innervation of the ciliary muscle [38,39]. As anticipated, age was found to be the single best predictor of AF demonstrating that the technique is a valid assessment of accuracy and amplitude of accommodative response which is known to decline with age [1]. No association was found between measures of AF and nLoA, nLoD, pLoA and pLoD. This is likely to be due to a number of factors; primarily, all of these metrics define the accommodative or disacommodative response rather than the gross accommodative/disaccommodative behaviour as assessed by AF. Furthermore, the latency metrics only evaluate the time taken to initiate the accommodative/disaccomodative response rather than the time taken to complete the response, which is more analogous to the AF measurements.

ToA, ToD, and oToAC all assess the time taken to complete the accommodative/disaccommodative response. Contrary to the assumption that oToAC relates better to AF as it provides a measure of the gross accommodative/disaccommodative response, out of all of the objective accommodative metrics, ToD was found to be the best predictor of AF and demonstrated the highest repeatability of all of the metrics. This observation may be explained by the slower response time of sToAC (derived from AF) relative to that of oToAC. A likely reason for these differences may be due to the objective measures of ToA, ToD, and oToAC accounting only for the time of response, whereas sToAC calculated from AF would reflect both time and accuracy of the accommodative response. sToAC is also influenced by the combined reaction times of the participant and the practitioner, which is not a factor in the objective measures. Furthermore, the subjectivity of the endpoint for sToAC and the influence of depth-of-focus are important factors that need to be considered.

In accordance with previous studies, all objective metrics examining accommodation were found to be slower than those assessing disaccommodation [22,40]. The rate of lenticular shape change during accommodation and disaccommodation appear to be asymmetric; with a more rapid increase in radius of curvature during disaccommodation. This difference in time for accommodation and disaccommodation may be related to the mechanism of accommodation. It has been suggested that disaccommodation occurs more rapidly due to the passive nature of increasing zonular tension, in contrast to accommodation which requires a decrease in zonular tension [22,40]. Alternatively, the nature of the accommodative response may be explained by the properties of the elastic capsule surrounding the lens. It is conceivable that the capsule has a viscoelastic nature rather than being purely elastic, causing a significant delay when generating a convex shape.

The Grand Seiko WAM auto-refractor took measurements of accommodation every 0.125 s, limiting all time and latency metrics measures to ±0.125 s and accounting for up to 19.8% of the total of the median of pLoD (0.63 s). This time interval is significantly greater than other studies that have examined the accommodative dynamic profile using power refractors, which have taken measurements at frequencies of 25 Hz (0.04 s) [4,8,23,24], or 30 Hz (0.03 s) [14]. The difference in frequencies of measurement acquisition compared to previous investigations [8,14,26,35,36,37] may partly explain the longer latency periods found in this study.

In contrast, the median ToA (2.09 s) and ToD (1.71 s) found in the present study are in accordance with the times observed in studies by Radhakrishnan, Allen, and Charman in 2007 (2.39 s and 2.04 s respectively) [25], and 2010 (1.89 s and 2.64 s, respectively) [4]. Other studies, which have measured accommodative response times have reported shorter times of between 0.53 s and 0.9 s [26,38,41,42], possibly due to inherent differences in the methodology and analysis.

## 5. Conclusions

To conclude, in line with previous studies, disaccommodation was noted to occur more rapidly than accommodation, and subjective metrics were found to overestimate the time taken for accommodative change in comparison to the objective measurements. Furthermore, the novel technique for calculating the latency of accommodation or disaccommodation was found to be faster and more repeatable than the methods used in previous studies; however, these metrics are not interchangeable. ToD was the most repeatable metric, and the accommodative metric that was most associated with AF.

## Figures and Tables

**Table 1:** The comparable subjective and objective tests for assessing the different parameters of accommodative function.

Parameters	Subjective Tests	Objective Tests
Time for the accommodative response	Accommodative facility	Accommodative dynamics (Latency of accommodation, Peak Velocity, Time Constant, Response Times)
Accuracy of response (Accommodative lag)	Binocular cross-cylinder	Accommodative dynamics (Lag) Dynamic retinoscopy
Absolute response or amplitude of accommodation	Push-up/Pull-down test Minus-to-blur/Defocus curves	Accommodative dynamics (Absolute Response/Magnitude of Response)
Sustainment of response	None applicable	Accommodative dynamics (Microfluctuations)

**Table 2:** The definitions and methods used to derive different time metrics from the accommodative dynamic profile.

Metric	Definition	Disparity in Methods Used to Define or Derive the Metric
Latency of accommodation (Figure 1: Red arrow)	The time delay between the onset of the accommodative stimulus and the initiation of the accommodative response.	Two methods have been used to define the end-point of this metric: Anderson et al., (2010) utilised the first data point where five consecutive data points demonstrated an increase in accommodation [14]. This requires visual inspection of the data by the observer. Other studies identified the initial point of a sequence where three consecutive data points increased in accommodation, followed by four data points where no two consecutive points decreased in accommodation [8,23,28].
Response times (Figure 1: Green arrow)	The time interval between the onset of the stimulus and reaching the maximum accommodative (or disaccommodative) response.	The exact methodology used to identify the precise start and end-points to calculate the time interval have not been stated in previous studies [4,24,26].
Time constant	The time taken for a set percentage of the total accommodative response to occur.	Different percentage points of the accommodative response have been used, including 63% [14,23,27]; however, Radhakrishnan and colleagues, defined the time interval as the period between reaching 10% and 90% of the total accommodative response [4,24].
Peak velocity	The maximum speed of the accommodative change reached at a set point of the accommodative response. The peak velocity is calculated using the following formula: $Vmax=\frac{a}{\tau }$ where *Vmax* is peak velocity, a is the accommodative response, and τ is the time constant.	Although studies tend to agree on this formula for calculation, variation in methods used to derive the time constant, ultimately lead to variations in the peak velocity calculated [4,14,23,24,27].

**Table 3:** The Intraclass correlation coefficients of each measured metric, showing both intra-observer repeatability and inter-repeatability.

	Intra-Observer Repeatability	Inter-Observer Repeatability
Accommodative Facility	0.843	0.889
Time for Accommodation	0.258	0.645
Time for Accommodation	0.811	0.568
Objective Time for Accommodative Change	0.491	0.575
Subjective Time for Accommodative Change	0.843	0.889
Novel Latency of Accommodation	0.937	0.384
Novel Latency of Disaccommodation	0.342	0.519
Previously used Latency of Accommodation	0.475	0.295
Previously used Latency of Disaccommodation	0.610	0.375
